# The CELF1 RNA-Binding Protein Regulates Decay of Signal Recognition Particle mRNAs and Limits Secretion in Mouse Myoblasts

**DOI:** 10.1371/journal.pone.0170680

**Published:** 2017-01-27

**Authors:** Joseph Russo, Jerome E. Lee, Carolina M. López, John Anderson, Thuy-mi P. Nguyen, Adam M. Heck, Jeffrey Wilusz, Carol J. Wilusz

**Affiliations:** 1 Department of Microbiology, Immunology and Pathology, Colorado State University, Fort Collins, Colorado, United States of America; 2 Program in Cell and Molecular Biology, Colorado State University, Fort Collins, Colorado, United States of America; Korea University, REPUBLIC OF KOREA

## Abstract

We previously identified several mRNAs encoding components of the secretory pathway, including signal recognition particle (SRP) subunit mRNAs, among transcripts associated with the RNA-binding protein CELF1. Through immunoprecipitation of RNAs crosslinked to CELF1 in myoblasts and *in vitro* binding assays using recombinant CELF1, we now provide evidence that CELF1 directly binds the mRNAs encoding each of the subunits of the SRP. Furthermore, we determined the half-lives of the *Srp* transcripts in control and CELF1 knockdown myoblasts. Our results indicate CELF1 is a destabilizer of at least five of the six *Srp* transcripts and that the relative abundance of the SRP proteins is out of balance when CELF1 is depleted. CELF1 knockdown myoblasts exhibit altered secretion of a luciferase reporter protein and are impaired in their ability to migrate and close a wound, consistent with a defect in the secreted extracellular matrix. Importantly, similar defects in wound healing are observed when SRP subunit imbalance is induced by over-expression of SRP68. Our studies support the existence of an RNA regulon containing *Srp* mRNAs that is controlled by CELF1. One implication is that altered function of CELF1 in myotonic dystrophy may contribute to changes in the extracellular matrix of affected muscle through defects in secretion.

## Introduction

The concept of RNA regulons, in which functionally related genes are co-regulated by specific RNA-binding proteins (RBPs), was first proposed by Keene and Tenenbaum in 2002 [1]. Since that time, the advent of RNA immunoprecipitation-based high throughput approaches including CLIP-seq [2] and PAR-CLIP [3] has facilitated the identification of large datasets of mRNA targets for a variety of RBPs, including CELF1 [4–8]. Additional studies have indicated that subcellular clustering of functionally related mRNAs through their interactions with RNA-binding proteins can result in a high local concentration of protein subunits to facilitate efficient macromolecular complex assembly [9]. Based on these ideas, altered function of a single RBP could have profound effects on the assembly/abundance of an entire multi-protein complex. Such regulation could be central to normal cellular responses, but might also be detrimental if the RBP were mutated or impaired by disease.

CELF1 (CUG-binding protein, ELAV-Like Family member 1) is an RBP which regulates gene expression at multiple steps, including splicing, polyadenylation, translation and/or mRNA decay [10,11]. CELF1 appears to be particularly important for skeletal muscle function as its over-expression in mice inhibits myogenesis [12], decreases muscular mass and function, and induces an array of muscular histological abnormalities [13]. Furthermore, several inherited human diseases affecting muscle display aberrant CELF1 expression or localization[14–16]. In the best studied example, Type 1 Myotonic Dystrophy (DM1), CELF1 is hyper-phosphorylated and over-expressed [14,17]. CELF1 over-expression in DM1 has been connected with pathogenic splicing abnormalities [18–21], but its impact on other steps of mRNA metabolism such as decay and translation is less understood.

We chose to focus here on a putative RNA regulon containing six mRNAs encoding protein subunits of the signal recognition particle (SRP)[22]. The SRP is a cytoplasmic ribonucleoprotein complex composed of the 7SL structural RNA and six different protein subunits (SRP9, SRP14, SRP19, SRP54, SRP68 and SRP72)[22]. The vast majority of SRP biogenesis occurs in the nucleolus, before export to the cytoplasm where the final subunit, SRP54, is incorporated [23,24]. The SRP binds the signal peptide in nascent secreted and membrane-bound proteins and induces translational stalling [25]. When SRP associates with its receptor in the endoplasmic reticulum (ER), translation resumes and the nascent protein is translocated into the ER lumen. We selected the SRP transcripts for further study because coordinated expression of SRP mRNAs might be necessary for efficient SRP complex assembly. Moreover, the secretory pathway is essential for formation of a functional extracellular matrix (ECM), and ECM components are frequently mutated/disrupted in muscular dystrophies [26]. In addition, autoantibodies to SRP subunits are a primary cause of inflammatory myositis [27]; a condition that can be difficult to distinguish from muscular dystrophy [28].

In this study, we investigated the effects of CELF1 expression on accumulation and decay of mRNAs encoding the SRP proteins, which we previously identified through RNA-immunoprecipitation [4]. We observed significant changes in the stability of *Srp* transcripts in response to CELF1 knockdown. This is likely directly due to loss of CELF1 as multiple experiments support direct binding of CELF1 to *Srp* transcript 3’UTRs. Furthermore, SRP19 and SRP68 proteins are over-expressed following CELF1 depletion. Our data support that this is sufficient to influence cellular processes dependent on the SRP as CELF1 depletion and SRP68 over-expression can each cause defects in cell migration, which relies on secretion of the ECM.

## Materials and Methods

### Cell culture and transfections

C2C12 cells (ATCC CRL1772) were grown at 37°C, 5% CO_2_ at or below 70% confluency in Dulbecco's Modified Eagle's Media (DMEM) containing 10% fetal bovine serum (FBS), penicillin (10 units/ml) and streptomycin (10 μg/ml). To create knockdown cell lines C2C12 myoblasts were infected using either a control lentivirus (LKO1) or a lentivirus encoding an shRNA against CELF1 (sh1739 Sigma MISSION ID clone NM_198683.1-1739s1c1), pools of cells were selected with puromycin (4 μg/ml) and CELF1 knockdown was then verified by western blot [29]. CELF1 expression was routinely reduced to less than 10% of control [4]. After the initial selection the stable cell lines were maintained in 1–2 μg/ml puromycin.

### Western blot analyses

30–50 μg of whole protein extract prepared by lysis of cells in RIPA buffer (50 mM Tris-Cl pH7.4, 150 mM NaCl, 0.5% deoxycholic acid, 1% NP-40, 1 mM EDTA, 0.05% SDS) were separated on 10% or 15% SDS-polyacrylamide gels and blotted onto polyvinylidene difluoride (PVDF) membrane. Suppliers, catalog numbers and concentrations for primary antibodies can be found in Table A in S1 File. In all cases, anti-mouse or anti-rabbit horseradish peroxidase-conjugated secondary antibodies (Santa Cruz Biotechnology) were employed as appropriate, and detection was by the SuperSignal Pico West kit (Pierce) using a Bio-Rad ChemiDoc imager and the ImageLab software for quantification.

### Gel shift analyses

Recombinant human GST-CELF1 protein was purified from *E*.*coli* as previously described [30]. α^32^P-UTP labeled transcripts were produced by *in vitro* transcription using SP6 or T7 RNA polymerase. Templates for transcription were obtained by PCR from murine cDNA with primers specified in Table B in S1 File. Each template contained the region indicated flanking the putative GU-rich CELF1 binding sites. The negative control template was a transcript derived from the 7SL RNA which does not contain CELF1-binding sites. Increasing concentrations of GST-hCELF1 protein were incubated with 3 fmol of RNA transcript and separated on a 5% native polyacrylamide gel as described previously [31].

### Formaldehyde cross-linking and RNA immunoprecipitation

RNA immunoprecipitation experiments were performed after formaldehyde cross-linking of myoblasts using a procedure based on that described previously [32]. Briefly, C2C12 cells were grown to ~60% confluency and cross-linking of RNA complexes was achieved by incubating the cells for 10 minutes in 1 X Phosphate Buffered Saline (PBS) containing 1% formaldehyde. Glycine (pH 7.0) was added to a final concentration of 250 mM and cells were rocked for 5 minutes to quench the reaction. Cells were washed three times in PBS and harvested by centrifugation. Cell pellets were resuspended in an equal volume of RIPA buffer and lysed by sonication. The RNA-CELF1 complexes were immunoprecipitated from cleared whole cell lysates by incubating with 2 μg of anti-CELF1 antibody (mAb 3B1; Santa Cruz Biotechnologies) or normal mouse IgG antibody on ice for 1 hour. Protein-G sepharose resin was added to precipitate bound complexes. Precipitates were washed twice each in NT2 (50 mM Tris-Cl pH 7.4, 150 mM NaCl, 1 mM MgCl_2_, 0.05% Nonidet P-40), RIPA, and High-Stringency RIPA (50 mM Tris-Cl pH 7.4, 1% NP-40, 0.1% Sodium Deoxycholate, 0.1% SDS, 1 mM EDTA, 1M NaCl, 1 M Urea, and 0.2 mM PMSF), for 10 minutes each. Crosslinks were reversed by incubation in crosslink reversal buffer (50 mM Tris-Cl pH 7.0, 0.5 mM EDTA, 10 mM DTT, and 1% SDS) for 40 minutes at 70°C. The RNA was isolated using TRIzol^®^ (ThermoFisher) extraction according to the manufacturer’s recommendations. cDNA was synthesized using random hexamers and Improm II Reverse Transcriptase (Promega) and subsequently used for PCR with primers specific for the 3’ UTR of the *Srp* mRNAs or for *Myc* mRNA as a negative control (see Table B in S1 File).

### Luciferase secretion assays

Control and CELF1 KD myoblasts were transfected using Lipofectamine^™^ 2000 (Life Technologies) [31] in a 96-well plate format with two reporter plasmids; one encoding a Firefly luciferase (FLuc; pLightSwitch_3’UTR from SwitchGear Genomics) and the other encoding a Gaussia luciferase (GLuc) bearing an N-terminal signal peptide [33]. Approximately 18 hr post-transfection, activity of the secreted GLuc in the media was measured by addition of the coelenterazine substrate (Nanolight Technology). Also, to account for variation in transfection efficiency, the FLuc activity in the cells was measured using the Steady Glo reagent (Promega). The signal from each luciferase was read in a Victor plate reader (Perkin Elmer). The GLuc reading was normalized to that of FLuc in each experiment.

### Analysis of mRNA abundance and half-lives

mRNA half-lives were determined as described previously [34] but using MTSEA-Biotin-XX rather than HPDP-biotin for conjugation [35]. Control (LKO1) and CELF1 KD cells were grown to ~60% confluency then incubated in 400μM 4-thiouridine (4sU; SIGMA). After 12 hours, total RNA was extracted via TRIzol^®^ (ThermoFisher) and treated with DNAse I. Total RNA was subjected to biotinylation as follows: 20–40μg of total RNA was spiked with total RNA extracted from *S*. *cerevisiae* that had been treated with 5mM 4-thiouracil for 5 min (see [36] for details) at a ratio of 5 μg C2C12 RNA to 1 μg of yeast RNA. The yeast RNA acted as an internal control for the biotinylation and fractionation process. Biotinylation was performed as described [35]. Briefly, 15μL of 10X biotinylation buffer (100mM HEPES [pH 7.5], 10mM EDTA) and 10μL 1mg/mL MTSEA-biotin-XX (Biotium) in 20% dimethylformamide were added to the RNA in a final volume of 150μl and incubated in the dark at room temperature with gentle agitation for 2 hours. After incubation, excess MTSEA-biotin-XX was removed by chloroform/isoamylalcohol extraction using Phase-Lock Gel Heavy 1.5mL tubes (5 PRIME). The RNA was precipitated and reconstituted in 105μL of ddH_2_0. The RNA was fractionated as follows: 50μL of RNA was bound to μMACS Streptavidin Microbeads (Miltenyi Biotech). Following two washes with high salt wash buffer (100 mM Tris-HCl (pH7.4), 10 mM EDTA, 1M NaCl and 0.1% Tween-20) the nascent (4sU-labeled) RNA was eluted from the column with 100mM DTT. All three fractions (Total, Flow-through/Pre-Existing and Nascent) were precipitated in the presence of glycogen, washed and resuspended in an equal volume of ddH_2_0. 1μL of total and nascent RNA were used to make cDNA using random hexamers and Improm II reverse transcriptase (Promega). Digital PCR (dPCR) was performed using primers specific to each SRP gene as well as yeast MFA2 as a control for biotinylation and fractionation using QX200 ddPCR EvaGreen supermix (Bio-Rad) according to the manufacturer’s recommendations. Total and nascent RNA copy numbers were normalized to the recovery for each sample (as determined by yeast MFA2 mRNA nascent/total ratio) to acquire abundances. Half-lives were determined using the equation t_1/2_ = t_L_ x ln(2)/ln(1-R), where t_L_ = labeling time and R = abundance in nascent RNA fraction/abundance in total RNA fraction. Labeling and fractionation were performed in triplicate, average half-lives were determined along with the standard error of the mean (SEM). A paired t-test (two-tailed) was performed to determine statistical significance. Abundances of *Srp* mRNAs were determined from the abundance in the total RNA fraction and normalized to levels of *Runx3* mRNA which was selected as an appropriate reference gene based on expression level and lack of variation between the Control and CELF1 KD cell lines. A paired t-test was used to determine statistical significance. Primers are described in Table B in S1 File. All primer sets were verified to perform with efficiencies of 90–110% and to generate a single product of the expected molecular weight.

### Over-expression of SRP68

The entire open reading frame of the murine SRP68 gene (*NM_146032*.*3*) was amplified by PCR using cDNA derived from C2C12 cells as a template. The resulting PCR product was digested with *Eco*RI and *Bam*H1 and cloned into the p3xFlag-CMV10 expression vector (Sigma). The primer sequences are shown in Table B in S1 File. The resulting construct or the empty vector were transfected into C2C12 cells as described above [31] and increased expression of SRP68 was verified by western blot 24 hrs later.

### Wound healing assays

These were performed essentially as described previously [37]. Equal numbers of control and CELF1 KD C2C12 myoblasts or control and SRP68 over-expressing myoblasts were allowed to reach near confluency in growth media. The tip of a pipette was used to scratch and remove the monolayer. The cells were washed twice with PBS buffer which was replaced with pre-warmed DMEM containing 0.1% FBS to minimize proliferation. The wound was imaged at 10X magnification. After incubation the cells were washed in PBS and reimaged. The number of cells that had migrated into the wound area was counted and the number in CELF1 KD or SRP68 over-expressing cells is represented as a percentage of those migrating in the control cells.

## Results

### CELF1 associates with Srp mRNAs

We previously used RNA-immunoprecipitation followed by microarray (RIP-Chip) to identify mRNAs associated with CELF1 [4]. Amongst the top 5% most enriched transcripts were several mRNAs encoding factors belonging to the secretory pathway, including three of the six protein subunits of the Signal Recognition Particle (*Srp54*, *Srp72* and *Srp68*). A more relaxed analysis (top 10% most enriched) suggested that *Srp9* and *Srp19* mRNAs were also significantly enriched in the CELF1 immunoprecipitate. In support of this, CELF1 CLIP-seq performed by others using mouse C2C12 cells identified binding sites in the 3’UTRs of *Srp9*, *Srp14* and *Srp72* mRNAs [5]. Both RIP-Chip and CLIP-seq have been used to identify RNA targets bound by CELF1 in HeLa cells and these also pinpointed *Srp* mRNAs as targets of CELF1 [8,38]. Thus multiple independent studies support that CELF1 binds to *Srp* mRNAs in human and mouse.

**Fig 1:**
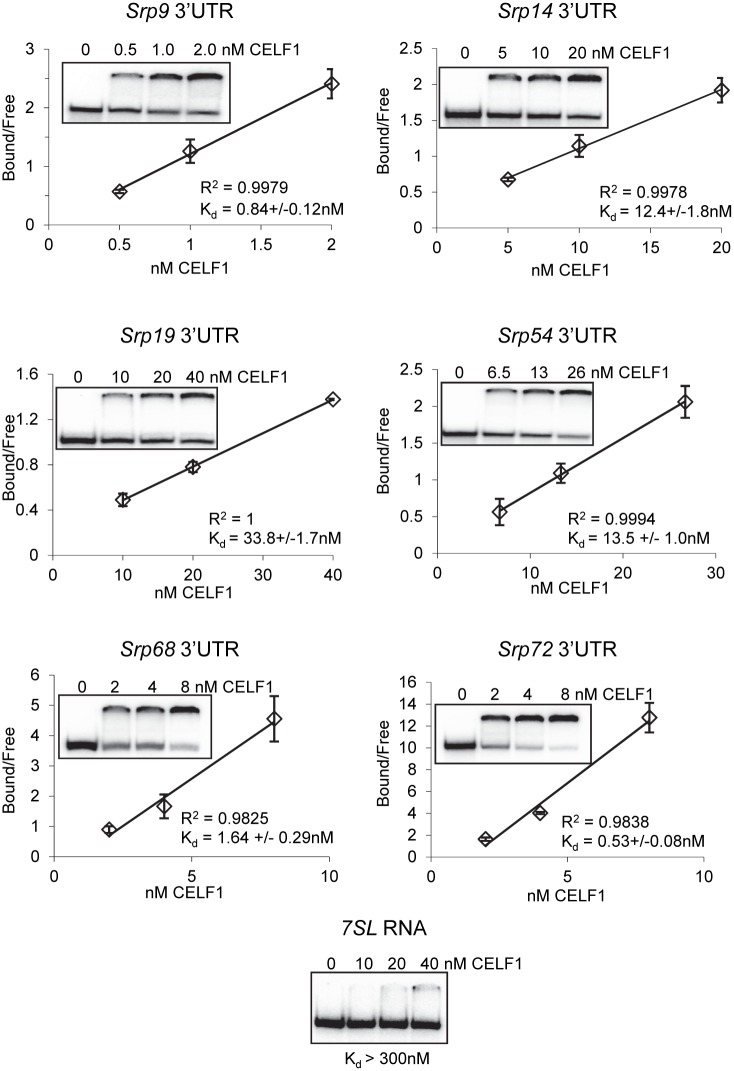
Sequence elements derived from the 3’UTRs of SRP mRNAs are bound by CELF1. Increasing concentrations of recombinant CELF1 were incubated with radio-labeled RNA fragments and separated on a native acrylamide gel. The proportion of RNA that bound to CELF1 was used to derive a dissociation constant.

Based on these observations, we hypothesized that CELF1 might play a role in coordinating expression of the SRP subunits to facilitate optimal assembly of the SRP complex. CELF1 binding through the 3’UTR can influence polyadenylation [39], translation [40,41], decay [11] and/or localization [42] of mRNAs, any of which could influence overall protein levels. We found GU-rich sequences resembling CELF1 binding sites in the 3’UTRs of all the *Srp* mRNAs (Table 1) supporting that the 3’UTR is the most likely binding site. In order to confirm this, we performed electrophoretic mobility shift assays, using recombinant human CELF1 protein and *in vitro* transcribed RNA fragments containing putative CELF1 binding site(s) derived from each of the *Srp* 3’UTRs. We found that all the 3’UTR fragments bound the recombinant protein with affinities ranging from 0.53 to 33.8 nM (Table 1 and Fig 1). In contrast, a fragment of the 7SL RNA, which contains no identifiable CELF1 binding sites, was not appreciably bound by CELF1 under the conditions employed (K_d_ > 300 nM (Fig 1)). We conclude that CELF1 is capable of binding the 3’ UTRs of all *Srp* mRNAs with high affinity *in vitro*. We cannot however rule out that CELF1 binds these mRNAs in additional locations *in vivo* as the complete 3’UTRs containing all possible binding sites were not tested, primarily due to limitations in separating protein complexes containing larger RNA fragments.

**Table 1:** CELF1 binding to 3’UTR of *Srp* mRNAs *in vitro*.

Gene	3’UTR length (nt)	Putative CELF1 binding sites			K_d_ (nM)
*Srp9*	960	GUGUUUUUUGUA	GUGUUGUC		0.84 ± 0.12
*Srp14*	380	UUGUGUU			12.4 ± 1.8
*Srp19*	371	UUUGUUUGUC			33.8 ± 1.7
*Srp54*	2475	UUCUGUGUUCUUGU	AGGUGUUUUUCUG		13.5 ± 1.0
*Srp68*	536	UUGUGUGUUUGUG	GUGUUUGUC	CUGUGUC	1.64 ± 0.29
*Srp72*	1566	GUGUGUGUGUAUUUGUG	UGUACCUUUGUUGUUUC		0.53 ± 0.08

GU-rich regions contained within each fragment that could act as CELF1 binding sites are shown. 3’UTR fragments ranging from 120–264 nt in length containing GU-rich elements were used for electrophoretic mobility shifts to derive the dissociation constants. Errors are standard deviations from three independent replicates.

**Fig 2:**
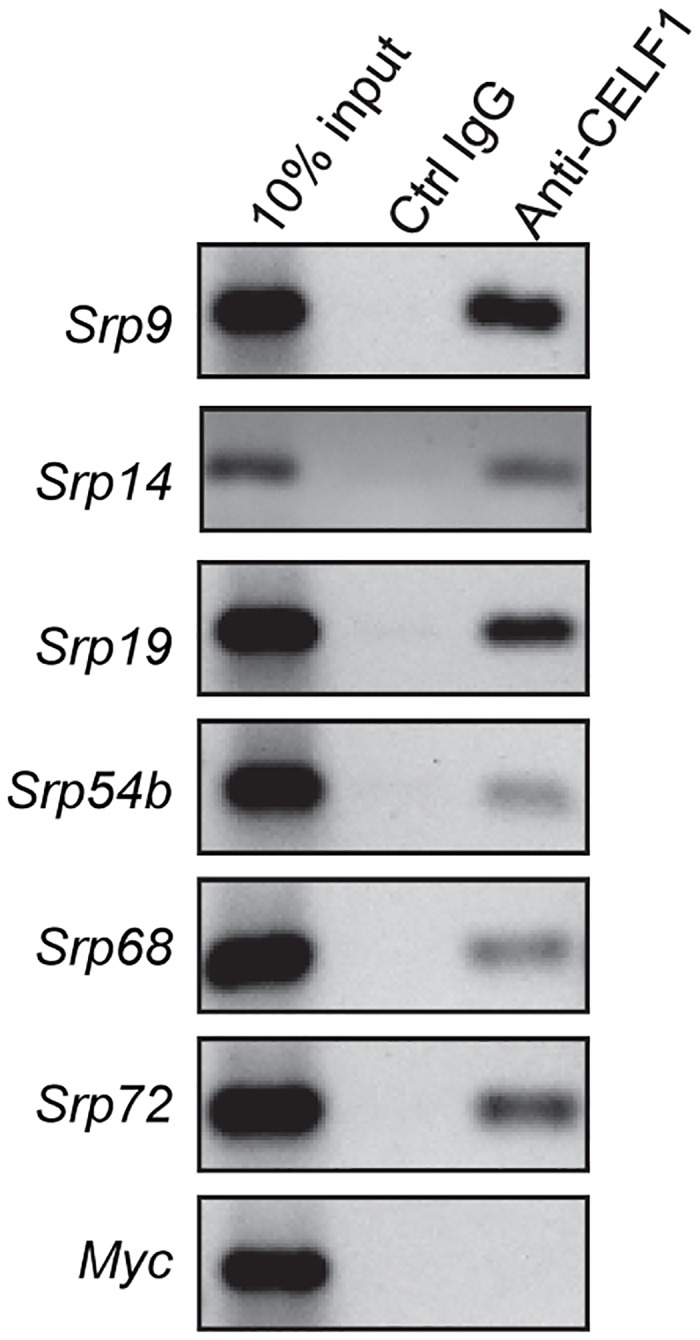
*Srp* mRNAs are associated with CELF1 in C2C12 myoblasts. C2C12 cells were treated with formaldehyde to stabilize protein-RNA interactions. Extracts were immunoprecipitated with anti-CELF1 antibody or a control IgG and Protein-G sepharose. RNAs associated with each immunoprecipitate were isolated and subject to reverse transcription and 35 or 42 (*myc*, *Srp14*) cycles of PCR. Products were separated on agarose gels and visualized by ethidium bromide staining.

The *in vitro* results were not entirely consistent with those obtained in our RIP-Chip experiment in that CELF1 appears to recognize the *Srp14* 3’UTR in our gel shift assays but this transcript was not enriched in the CELF1 immunoprecipitate. One possible explanation is that the interaction was lost during the immunoprecipitation. We therefore repeated the RNA immunoprecipitation but this time using protein extracts from cells that had been cross-linked with formaldehyde prior to cell lysis to stabilize protein/RNA interactions during the isolation and immunoprecipitation. A control immunoprecipitation using normal IgG was also performed in parallel. RT-PCR was then used to detect CELF1-associated transcripts in the immunoprecipitate. As shown in Fig 2, all six *Srp* transcripts were readily detected in the anti-CELF1 immunoprecipitate but not in the control IgG immunoprecipitate. As expected, a control transcript that does not contain GU-rich elements, *Myc*, was not associated with CELF1. These results demonstrate that CELF1 can associate with all the *Srp* mRNAs in living cells, but binding to *Srp14* mRNA may be more easily disrupted than the other associations as it was not observed without cross-linking [4].

### CELF1 depletion stabilizes Srp mRNAs

**Fig 3:**
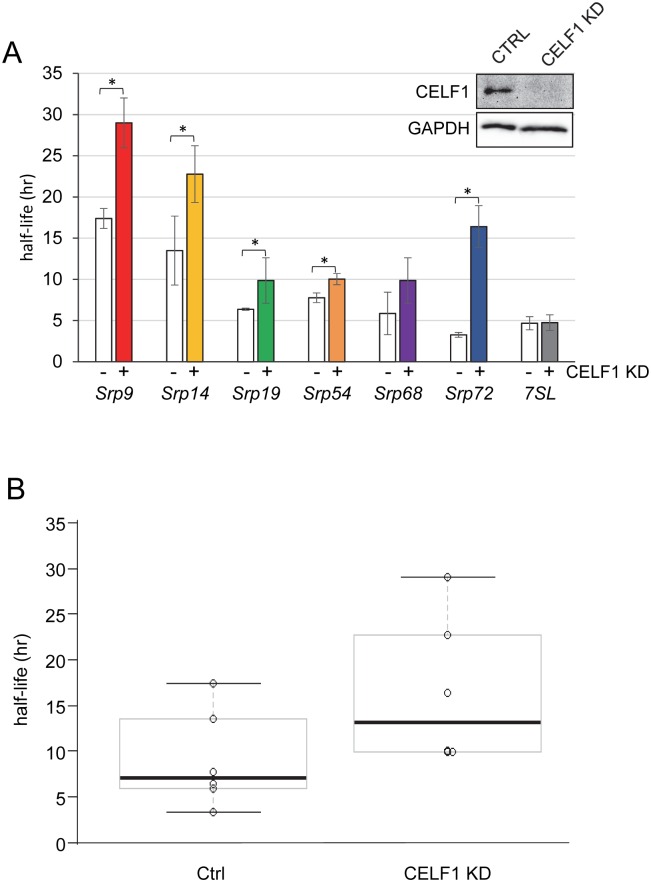
Srp mRNAs are stabilized following CELF1 knockdown. A: Control (white bars) and CELF1 KD (filled bars) C2C12 cells were treated with 4sU and total RNA was isolated at 12 hours. Following biotinylation and fractionation, the abundance of each *Srp* mRNA in total and labeled fractions was determined by dPCR and used to derive the half-life. The results were derived from three independent experiments for all except Srp54 and 7SL RNAs which were derived from two experiments. The error bars represent the standard error of the mean. Asterisks denote p<0.05. The inset shows a western blot verifying that CELF1 protein was effectively undetectable in CELF1 KD cells, GAPDH was evaluated as a loading control. B: *Srp* mRNA half-lives in Control and CELF1 KD C2C12 cells derived from the graphs in (A) are plotted for comparison.

We next wanted to determine the consequences of CELF1 binding on *Srp* mRNA decay. We were able to use data generated from our previous global analysis of mRNA decay rates in C2C12 cells [4] to estimate the normal half-lives for each of the *Srp* mRNAs. The calculated mRNA half-lives ranged from 2.7 hr for *Srp68* to almost 6 hr for *Srp72* (Fig A in S1 File). The standard approach to assay mRNA decay rates, used in our previous study, involves inhibition of transcription with Actinomycin D (ActD). This method is less than ideal when assaying relatively stable mRNAs due to the toxicity of prolonged treatment [43,44]. Therefore, we adopted an assay based on the incorporation of 4-thiouridine (4sU) into nascent transcripts, which has fewer and less drastic effects on cell health [34,35]. Briefly, Control and CELF1 KD cells were treated with 4sU for 12 hours and total RNA isolated. Upon addition of 4sU (t = 0 min), unlabeled transcripts cease to accumulate and the change in their abundance at subsequent time points reflects their rate of decay. The newly synthesized 4sU-containing transcripts were conjugated to biotin and enriched by passing over streptavidin-agarose. We measured the abundance of each S*rp* mRNA in total RNA before fractionation and in the labeled fraction using the ratio to derive a half-life for each transcript (see Materials & Methods for equation and additional details). In control cells, the half-lives for all the transcripts, except *Srp72*, were longer than those derived using ActD treatment (Fig B in S1 File) supporting the notion that ActD has unwanted and unpredictable effects on mRNA decay [45,46]. *Srp19*, *Srp54*, *Srp68 and Srp72* all had half-lives in a similar range (3.3–7.8 hr) while *Srp9* and *Srp14* were considerably more stable (17.4 and 13.5 hr respectively) (Fig 3A and Fig B in S1 File). These half-lives were similar to those derived by 4sU labeling in murine NIH 3T3 fibroblasts [47], except for *Srp9* which appears significantly more stable in C2C12 cells than in fibroblasts (Fig B in S1 File).

When we compared the half-lives in the control cells to those in the CELF1 KD myoblasts, we found that all transcripts except for *Srp68* mRNA showed significant increases in half-life in the CELF1 KD cells (Fig 3A). As shown in Fig 3B, both the mean half-life and the range of half-lives for all *Srp* mRNAs are greater in the CELF1 KD cells than in the controls. At the same time, we evaluated the decay of the RNA subunit of the SRP complex, 7SL RNA, which is not directly associated with CELF1. We found that the decay of 7SL RNA is not affected by CELF1 KD (Fig 3A).

**Fig 4:**
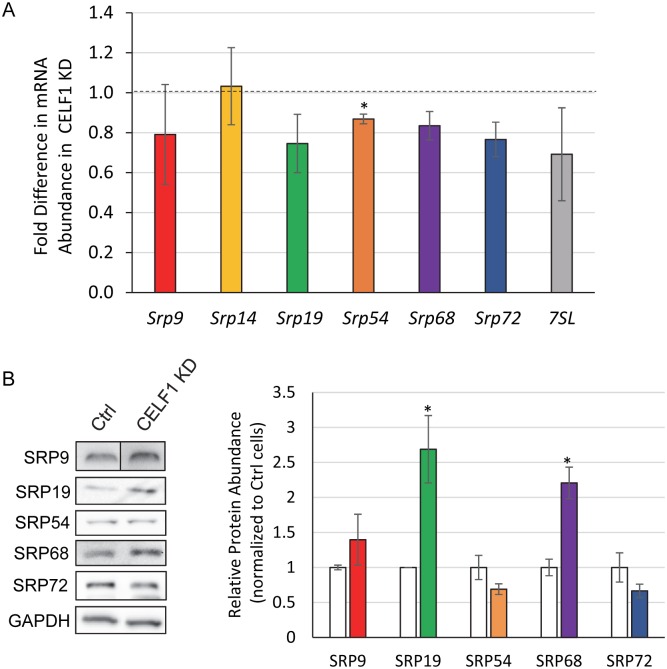
SRP protein expression is affected in CELF1 KD cells. A: Abundance of *Srp* mRNAs was determined by dPCR and normalized to *Runx3* mRNA abundance. The fold change in abundance of each *Srp* RNA in CELF1 KD cells is shown. Error bars represent the standard error of the mean for three independent replicates. Statistical significance was determined by paired t-test. The asterisk denotes p<0.05. B: Abundance of SRP proteins in whole cell lysates from Control and CELF1 KD cells was assessed by western blotting (left panel). For quantification, the abundance of GAPDH was used for normalization. In the graph, (right panel) SRP protein abundances in CELF1 KD (colored bars) are shown relative to the abundance in the Control (Ctrl) cell line (white bars). Error bars represent the standard error of the mean derived from three independent experiments. Statistical significance was determined using the t-test. Asterisks indicate p<0.05.

We also examined the abundance of *Srp* mRNAs following CELF1 KD but did not see any dramatic differences (Fig 4A). On the surface this is surprising given that some of the transcripts show large changes in mRNA decay rate (Fig 3A). However, we and others have found that the effects of mRNA decay on abundance are not generally predictable [37,48,49]. This phenomenon has been attributed to the existence of poorly characterized buffering mechanisms that alter transcription rates to compensate for undesirable changes in mRNA decay [50–52] and to effects of RBP depletion on expression of transcription factors [48].

We next wanted to assess whether the effect of CELF1 on *Srp* mRNA stability results in an effect at the protein level. We measured SRP protein levels by western blot in control and CELF1 KD myoblasts and found the levels of SRP19 and SRP68 proteins were reproducibly increased over two-fold in the CELF1 KD cells (Fig 3B). The abundance of the other three SRP proteins that we were able to identify effective antibodies for was not significantly altered. Therefore, the relative levels of the SRP subunits are out of balance in the CELF1 KD cells, with SRP68 and SRP19 present in excess.

### CELF1 depletion results in enhanced protein secretion

**Fig 5:**
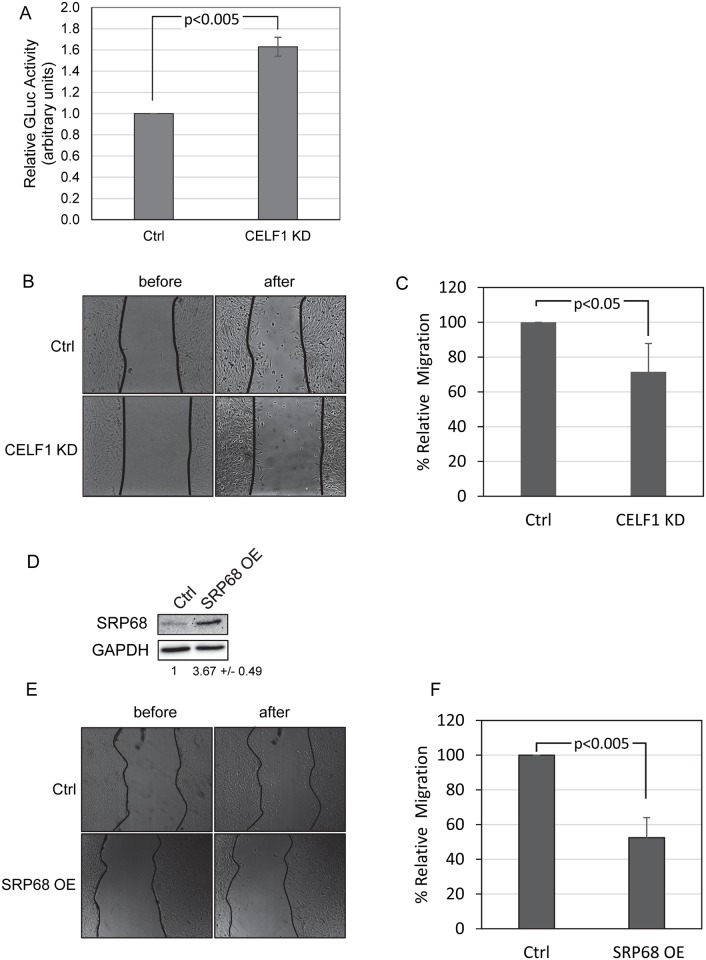
CELF1 KD cells have increased secretory capacity and perform poorly in wound healing assays. A: Control (Ctrl) and CELF1 KD cells were co-transfected with constructs encoding secreted and cytoplasmic luciferase proteins. Gaussia luciferase activity in the media was normalized to cytoplasmic firefly luciferase activity. Errors represent the standard deviation from three independent experiments. B: Control (Ctrl) and CELF1 KD cells were grown to ~90% confluence and then displaced by scratching. The cells were imaged immediately and again after incubation. C: The number of cells migrating into the wound is represented as a percentage of the number in the control cells. The error bars represent the standard deviation from three experiments. D: Western blot demonstrating that SRP68 is over-expressed following transfection of a construct encoding the murine SRP68 protein. E: Following transfection of the SRP68 over-expression construct or a control empty vector, wound healing assays were performed as described for B. F: Wound healing was quantified as for C.

Our results point to CELF1 having an important role in regulating the overall abundance of the SRP subunits. We therefore examined whether there was an effect on the overall secretory capacity of cells depleted of CELF1 using a Gaussia luciferase (GLuc) that bears a signal peptide and is efficiently secreted into the media [33]. We transfected control and CELF1 KD myoblasts with plasmids encoding the secreted GLuc and a cytoplasmic Firefly luciferase (FLuc). The next day, the media was assayed for GLuc activity and the cells were lysed and assayed for FLuc activity. GLuc activity was then normalized to FLuc to control for differences in transfection efficiency. We found GLuc was reproducibly secreted more efficiently in the CELF1 KD cells (Fig 5A). Similar results were obtained in CELF1 KD cells expressing an shRNA targeting a different region of the CELF1 mRNA, verifying that the effect on secretion is specific to CELF1 KD (data not shown).

### CELF1 depletion impairs wound healing

Production of the extracellular matrix (ECM) is exquisitely dependent on the secretory pathway[53]. We therefore hypothesized that cellular functions such as cell migration, which relies heavily upon the cell interactions with the ECM, might be affected in CELF1 KD cells. We used a wound healing assay to investigate. Briefly, Control and CELF1 KD cells were grown to near confluency and then scratched to remove the monolayer. The cells were incubated in low serum media to reduce proliferation and healing was assessed by counting the number of cells migrating into the wound after six hours. We observed that CELF1 KD cells reproducibly performed poorly compared to controls in this assay (Fig 5B and 5C). This result is consistent with our finding of enhanced secretion in CELF1 KD cells; alterations in the levels of secreted ECM proteins and membrane bound receptors are expected to modulate cell-matrix interactions that influence migration [54].

### Over-expression of SRP68 impairs wound healing

If the defects in wound healing in CELF1 knockdown cells are connected with SRP subunit imbalance, then increasing abundance of SRP subunits by other mechanisms should be sufficient to induce a similar phenotype. Indeed, when we transfected C2C12 cells with plasmid encoding Flag-SRP68 protein we were able to detect a reduction in cell migration (Fig 5D–5F) similar to that induced by CELF1 depletion. This suggests that SRP68 may be a limiting factor in assembly of functional SRP.

## Discussion

In this study, we demonstrated that the CELF1 RNA-binding protein can specifically associate with sequences in the 3’UTR of each of the SRP mRNAs *in vitro* and in cultured myoblasts. Depletion of CELF1 resulted in stabilization of five SRP mRNAs and increased abundance of SRP19 and SRP68 proteins. Depletion of CELF1 also induced phenotypes consistent with disruption of the secretory pathway: CELF1 KD cells exhibited an increased ability to secrete a luciferase protein and slower migration in a wound healing assay. Finally, the defects in cell migration could be recapitulated by over-expression of SRP68, independent of CELF1 knockdown.

Our results demonstrate that CELF1 can bind to the 3’UTRs of all six SRP mRNAs, and influences the rate of decay for at least five SRP transcripts (*Srp9*, *Srp14*, *Srp19*, *Srp54* and *Srp72*) as well as the abundance of SRP19 and SRP68 proteins. Taken together, these data support that the SRP complex relies on CELF1 to maintain an appropriate balance of the individual subunits, however the overall impact of CELF1 binding may be different for each transcript. In part, this may reflect the affinity of CELF1 for the 3’UTR of each transcript—CELF1 had the highest affinity for the *Srp72* 3’UTR sequences (Table 1), and this mRNA was the most stabilized following loss of CELF1 (Fig 2A). However, the position of the CELF1 binding site with respect to other elements such as miRNA binding sites, binding sites for other RBPs may also play a role. For example, HuR and MBNL1 proteins are known to compete with CELF1 for binding and regulation of some RNA substrates [7,40,41]. Such competition might mask the effects of CELF1 knockdown under conditions where binding of another factor prevails.

Although we might have predicted that SRP subunits would have similar protein abundances in normal cells as they form a single complex, proteomics data from myotubes and skeletal muscle suggest that this may not in fact be the case (Fig C in S1 File [55]). It is possible that the more abundant subunits have other functions in addition to being components of the active SRP. For example, in human cells, SRP9 and SRP14 are present in excess and form a complex with the primate-specific Alu RNA [56]. In addition, fragments of 7SL RNA generated by Dicer cleavage may associate with a subset of SRP subunits and influence their association with the full length 7SL [57,58]. Alternatively, the limiting subunits may be upregulated under certain conditions to allow increased secretory capacity, or some subunits may be incorporated into the SRP less efficiently requiring that they be present at a higher concentration. At the protein level, we found that SRP19 and SRP68 were elevated following CELF1 KD. If association of these two subunits were inefficient, then increasing their concentration could enhance complex assembly and increase the overall abundance of functional SRP. In support of this idea, we found that over-expression of SRP68 is sufficient to impair cell migration, consistent with an increase in secretion of ECM components.

Association of SRP mRNAs with CELF1 potentially allows them to be regulated in a coordinated fashion by any stimuli that alter CELF1 abundance or activity by influencing translation, turnover and/or post-translational modification of CELF1. To our knowledge, natural conditions that result in altered levels of SRP proteins have not been identified, but conditions that influence CELF1 activity have been described. For example, during T cell activation, CELF1 is phosphorylated leading to reduced binding to its targets [59]. The target RNA regulon in this case encodes proteins necessary for the transition out of quiescence and CELF1 inactivation therefore allows rapid upregulation in response to extracellular signals. CELF1 activity is also disrupted in DM1, where the protein is hyper-phosphorylated and over-expressed [17]. The precise effect on CELF1 function and activity remains unclear, but evidence suggests that the splicing activity of CELF1 is elevated in DM1 [7,13] supporting that in this case the protein can maintain its association with RNA. Thus in DM1 it is possible that over-expression of CELF1 could destabilize target transcripts, including the *Srp* mRNAs.

We note that the stabilization of *Srp* mRNAs in CELF1 knockdown cells has little influence on the abundance of these mRNAs at steady state (Fig 4A). It is important to note that changes in mRNA stability have the biggest impact during a cellular response. More stable mRNAs take longer to reach a new steady state following induction or repression of transcription [60]. This means that the response to a stimulus will be slower. Thus, the biological impact of stabilizing CELF1-regulated mRNAs may be exacerbated under conditions where target transcripts are normally up- or down-regulated.

The association of CELF1 with mRNAs encoding components of the secretory pathway is intriguing, given that muscle is exquisitely dependent on secretion both for deposition of a functional extracellular matrix to transduce its contractile activity, and for myokine signaling. Moreover, defects in muscular ECM components are connected with various muscular dystrophies [26]. Our results show that knockdown of CELF1 enhances secretion of a reporter and slows migration in C2C12 cells. It seems possible therefore that the mis-regulation of CELF1 in DM1 may contribute to pathogenesis by disrupting secretion of ECM and/or other extracellular proteins resulting in a muscular dystrophy despite the fact that the gene affected in DM1, DMPK, does not encode an ECM component. Whether the secretion defect seen in CELF1 KD cells is directly connected to regulation of secretory pathway mRNAs or through another mechanism will require further investigation, but the fact that over-expressing SRP68 protein induces similar phenotypes is certainly consistent with this idea.

## Supporting Information

S1 File Supporting information. This file contains Fig A-C and Tables A and B as supporting information.
(PDF)
