# Supplementary material for: The CELF1 RNA-Binding Protein Regulates Decay of Signal Recognition Particle mRNAs and Limits Secretion in Mouse Myoblasts
Source: PLoS One. 2017 Jan 27;12(1):e0170680. doi: 10.1371/journal.pone.0170680 (PMC5271678; doi:10.1371/journal.pone.0170680)
Supplement: S1 File — This file contains Fig A-C and Tables A and B as supporting information. (PDF) [file pone.0170680.s001.pdf]

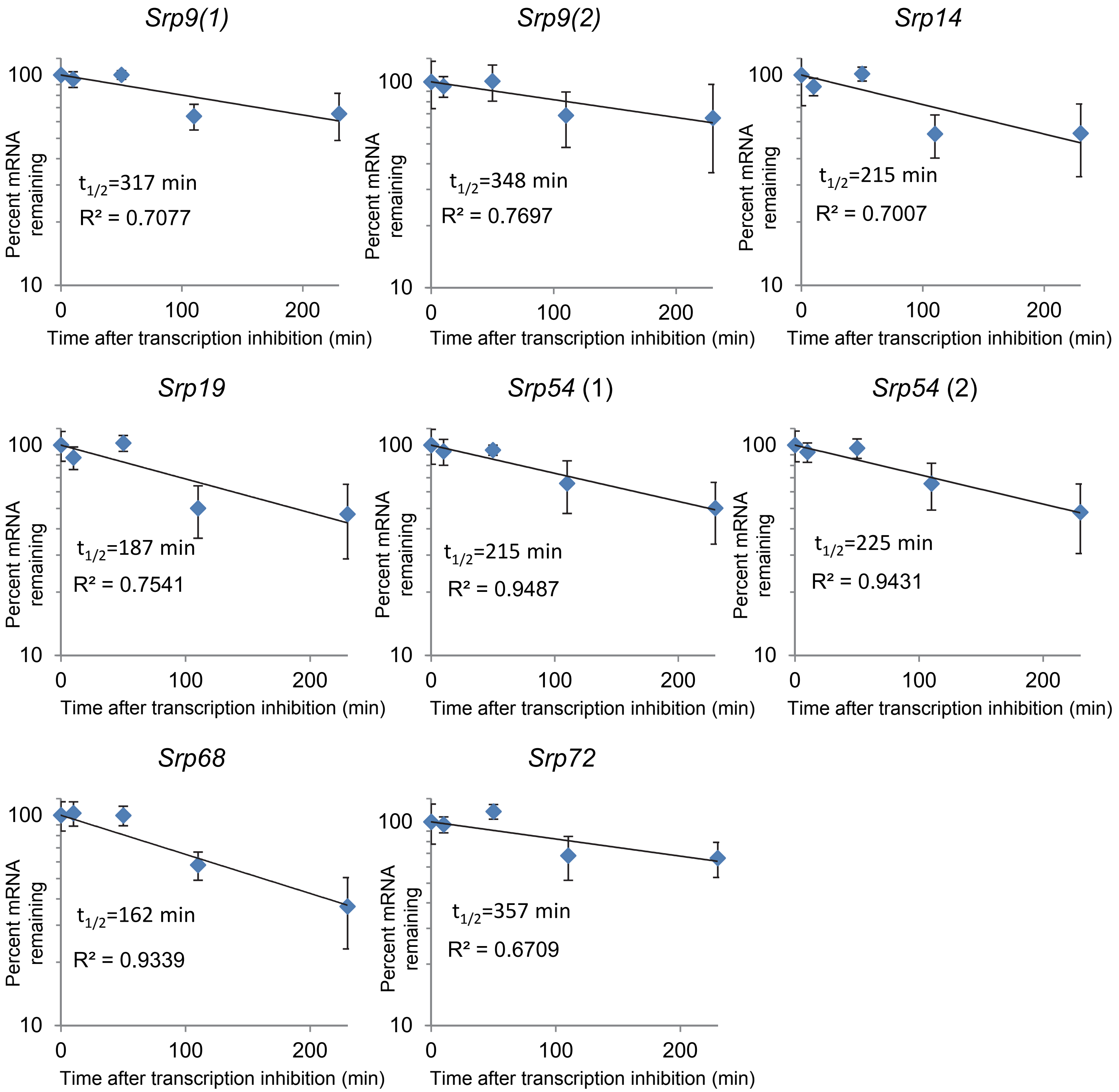


**Figure A: Half Lives of Srp mRNAs derived by microarray analysis following ActD treatment.** Changes in mRNA abundance following inhibition of transcription were plotted over time. Abundances at each time point represent the mean of three biological replicates and were derived from our previously published microarray experiment (ref 4). Error bars represent the standard deviation. For Srp9 and Srp54 two different transcripts were detected and their half-lives are plotted separately.

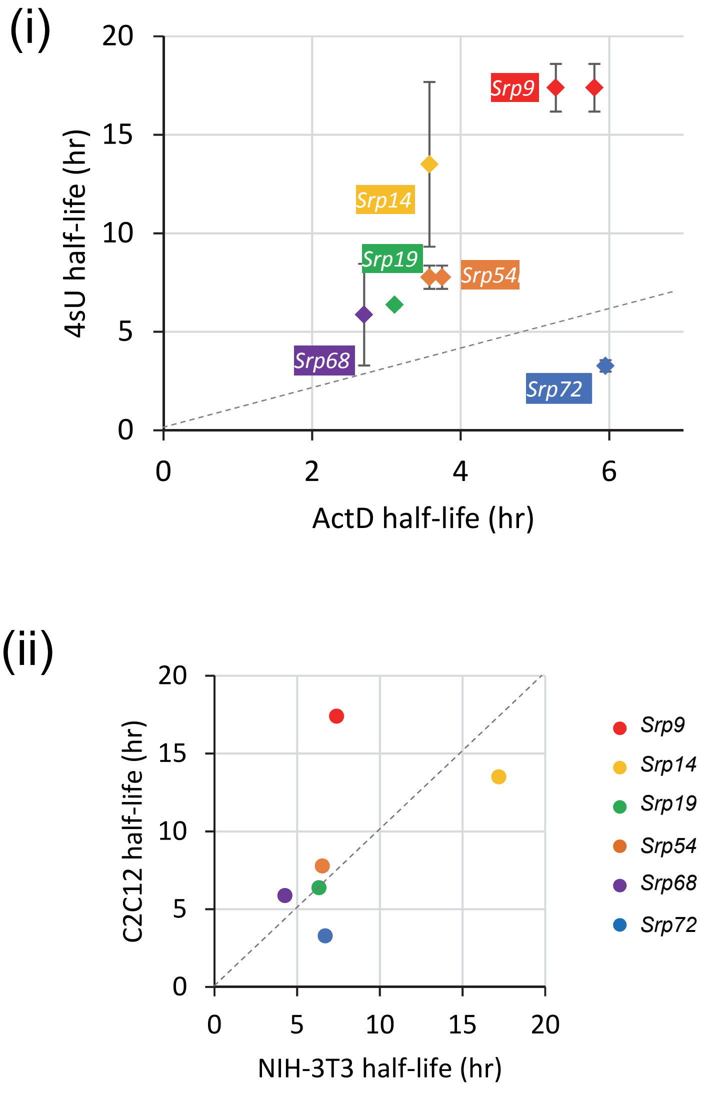


**Figure B: (i)** Half-lives derived from 4sU labeling are generally longer than those measured following Actinomycin D treatment. The dashed line indicates perfect correlation between the two approaches. Srp9 and Srp54 each encode two transcripts that had different half lives by ActD/microarray but cannot be distinguished by the 4sU approach. Error bars represent the SEM from three independent biological replicates. (ii) Comparison of Srp mRNA half lives in C2C12 cells (this study) and murine NIH-3T3 fibroblasts (ref 47). Decay was assessed through 4sU labeling in both cases. The dashed line indicates perfect correlation between the two cell types.

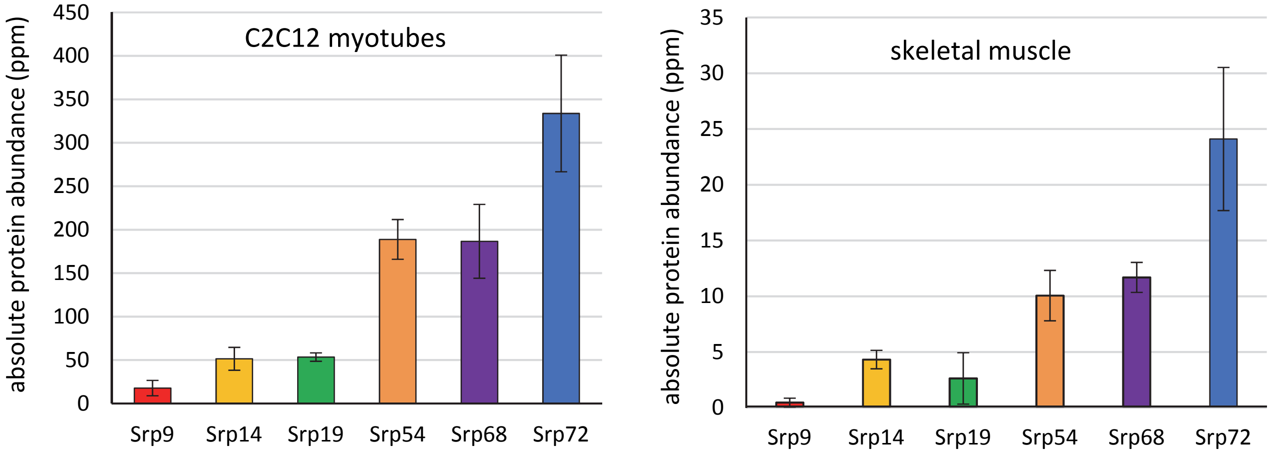


**Figure C: SRP proteins have a range of abundances in myotubes and skeletal muscle.** Abundances are plotted in parts per million and are derived from data generated through mass spectrometry-based proteomic analysis by Deshmukh et al, 2015 (55).

Table A: Antibodies used in this study

| Antigen | Description | Company & Catalog # | Dilution |
|---|---|---|---|
| SRP9 | Rabbit Polyclonal | Proteintech 11195-1-AP | 1:5,000 |
| SRP19 | Rabbit Polyclonal | Proteintech 16033-1-AP | 1:2,000 |
| SRP54 | Mouse Monoclonal $IgG_1$ | Santa Cruz sc136303 | 1:5,000 |
| SRP68 | Rabbit Polyclonal | Proteintech 11585-1-AP | 1:2,500 |
| SRP72 | Rabbit Polyclonal | Sigma HPA034621 | 1:1,000 |
| CELF1 | Mouse Monoclonal $IgG_1$ | Santa Cruz sc-20003 (3B1) | 1:500 |
| GAPDH | Mouse Monoclonal $IgG_{2b}$ | Chemicon mAB374 | 1:20,000 |

**Supplementary Table B:** Oligonucleotide primers used in this study.

| Gene<br>(Genbank Accession#) | Primer Sequences | Product size (nt) |
|---|---|---|
| Primers for RT-PCR and qRT-PCR | | |
| *Srp9* (NM_012058.3) | Srp9F 5′ TCCTGTGGTGTGAAGAGCAG 3′<br>Srp9R 5′ GCACACATAACTGCGTCTCG 3′ | 126 |
| *Srp14* (NM_009273.4) | Srp14F 5′ TCCTCTGTTTGCTGCTTTCA 3′<br>Srp14R 5′ ATATCCAGCCACACAGCACA 3′ | 137 |
| *Srp19* (NM_025527.3) | Srp19F 5′ ACATGGCATCCTTGTGTCTG 3′<br>Srp19R 5′ AAGCTGGATGCAAACTTCTGA 3′ | 129 |
| *Srp54*[†] (NM_011899.4) | Srp54F 5′ AAAATAAGGCCGTAGCAGCA 3′<br>Srp54R 5′ ACCGTCACTGGAATTGAAGC 3′ | 98 |
| *Srp68* (NM_146032.3) | Srp68F 5′ CCCATGGATTTGAAATGACC 3′<br>Srp68R 5′ CTGACCACAACTCTGGGTGA 3′ | 102 |
| *Srp72* (NM_025691.1) | Srp68F 5′ TCACAAGGCTTTCCTGCTCT 3′<br>Srp68R 5′ AGCAATGTCGACCCCTCTTA 3′ | 96 |
| *Gapdh* (NM_008084) | GapdhF 5′ TCACCACCATGGAGAAGGC 3′<br>GapdhR 5′ GCTAAGCAGTTGGTGGTCA 3′ | 168 |
| *Myc* (NM_001177352.1) | MycF 5′ CTGTCCATTCAAGCAGACGA 3′<br>MycR 5′ TCCAGCTCCTCCTCGAGTTA 3′ | 138 |
| *Runx3* (NM_019732.2) | Runx3F 5′ ACTTCCTCTGCTCCGTGCT 3′<br>Runx3R 5′ GGTCACCACCGTTCCATC 3′ | 107 |
| *Gluc* (plasmid sequence) | GLucF 5′ TGAGATTCCTGGGTTCAAGG 3′<br>GLucR 5′ GTCAGAACACTGCACGTTGG 3′ | 121 |
| *Fluc* (plasmid sequence) | FLucF 5′ CACCTTCGTGACTTCCCATT 3′<br>FLucR 5′ TGACTGAATCGGACACAAGC 3′ | 168 |
| *MFA2* (NM_001182983.1 ) | MFA2F 5′AATGCAACCGATCACCACTG 3′<br>MFA2R 5′GATAACACAGGCGGGATCCC 3′ | 117 |
| *Srp68* (NM_146032.3) | SRP68ORF-F 5′ *GATCGAATTCA*GCTGCTGAGAAGCAGATCCCG 3′<br>SRP68ORF-R 5′ *TCGCGGATCC*TTAGCTCCTGAAACCAAAGATG 3′ | 1898 |
| Primers to generate products for *in vitro* transcription of 3'UTR sequences<br>(forward primers have the T7 or SP6 promoter at the 5' end) | | |
| *Srp9* (NM_012058.3) | 9-IVT-F 5′ TAATACGACTCACTATAGGGTAGCATGCCTCTCAGAACTG 3′<br>9-IVT-R 5′ ATTTCAAGTTATTCACCATGAC 3′ | 191 |
| *Srp14* (NM_009273.4) | 14-IVT-F 5′ TAATACGACTCACTATAGGGATTCCAGCCGTAAGGTTTC 3′<br>14-IVT-R 5′ CCACCTGTAGGATAACACAAC 3′ | 212 |
| *Srp19* (NM_025527.3) | 19-IVT-F 5′ TAATACGACTCACTATAGGGAGAGACATGGCATCCT 3′<br>19-IVT-R 5′ CATTGTTAACTGTTGATGAAGATGG 3′ | 120 |
| *Srp54* (NM_011899.4) | 54-IVT-F 5′ TAATACGACTCACTATAGGGTGATGCCAGTGAAAGAATGG 3′<br>54-IVT-R 5′ TTGAGGTGGGAAGACACATAC 3′ | 189 |
| *Srp68* (NM_146032.3) | 68-IVT-F 5′ TAATACGACTCACTATAGGGCAGAGCTCAGCACCCTCAG 3′<br>68-IVT-R 5′ AATCCATGGGACTGAGGACA 3′ | 264 |
| *Srp72* (NM_025691.1) | 72-IVT-F 5′ TAATACGACTCACTATAGGGCCAGAAACTCACCGTCTA 3′<br>72-IVT-R 5′ AGAACACTGGGGCAAGAGTC 3′ | 236 |
| 7SL RNA* | 7SL-IVT-F 5′ CATAGCATTTAGGTGACACTATGGCCGGGCGCGGTGGCGCG 3′<br>7SL-IVT-R 5′ AGAGACGGGGTCTCGCTATGTTGCCC 3′ | 322 |

[†]Note that the primers used to detect Srp54 detect the mRNA products from all three murine Srp54 paralogs (*Srp54a, Srp54b* and *Srp54c*) which encode virtually identical proteins.
*The murine 7SL RNA is not annotated in Genbank. We used the human 7SL RNA sequence (NR_002715.1) and blasted it against the mouse genome to identify a region on Chromosome 12 with 99% identity to the human gene. This region was used to design the 7SL primers.
